# Mitral regurgitation recovery and atrial reverse remodeling following pulmonary vein isolation procedure in patients with atrial fibrillation: a proof of concept cardiac MRI study

**DOI:** 10.1186/1532-429X-14-S1-P210

**Published:** 2012-02-01

**Authors:** Sahadev T Reddy, William Belden, Mark Doyle, Diane A Vido, June Yamrozik, Ronald B Williams, Robert W Biederman

**Affiliations:** 1 Allegheny General Hospital, Pittsburgh, USA

## Summary

Atrial reverse remodeling triggered by favorable pulmonary vein isolation for atrial fibrillation results in regression of mitral regurgitation.

## Background

Atrial fibrillation (AF) is a chronic and progressive disease, and if uncontrolled results in permanent remodeling of the myocardium. Reverse remodeling of the left atrium (LA) after the successful pulmonary vein isolation (PVI) in recurrent/chronic AF may occur, however, mitral regurgitation (MR) recovery after the successful PVI has not been demonstrated but would be an important factor in PVI durability.

## Objective

Retrospectively analyze the effectiveness of pulmonary vein isolation (PVI) procedure in patients with AF for evidence of atrial reverse remodeling and recovery of MR via CMR.

## Methods

Prior to PVI, patients underwent a clinically indicated CMRI, post-PVI (6±1months) patients underwent a follow up MRI, and were classified into 2 groups: responders (R) to PVI, non responders (NR) to PVI as assessed by cessation of AF at the end of 3 month blanking period. Further, CMR was used to evaluate the severity of MR (0 to 4+) and to relate changes in MR to LA, LAA volumes as well as mitral apparatus (mitral annulus, tenting area, tenting height and tenting angle). For continuous variables, group differences were assessed by unpaired or paired two-sample t tests or their non-parametric equivalents. We used chi-square tests for evaluating categorical variables.

## Results

Of the 94 patients with AF who underwent PVI, 76 (81%) were classified as R and 18 (19%) were classified as NR. Mean age, mean BSA and antiarrhythmic therapies were similar between the groups. MR pre vs. post in the R group significantly improved (Mean 0.78, Median 1.0 vs. Mean 0.51, Median 0, P 0.01) and was matched by favorable mitral geometry reverse remodeling [annulus (34.5 ± 3.9 mm vs. 32.6 ± 3.9 mm, p <0.001), tenting area (169.7 ± 55.9 mm2 vs. 139.0 ± 40.6 mm2, p < 0.001), and tenting height (8.0 ±2.0 mm vs. 7.2 ± 1.8 mm, p <0.001), tenting angle (128° ± 11° vs. 130° ± 10°)]. However, in the NR group MR failed to improve (Mean 1.06 Med 1.0 vs. Mean 0.67 Med 0, p NS), and paralleled failure of mitral geometry reverse remodeling [annulus (33.1 ± 4.0 mm vs. 32.9 ± 3.6 mm, p NS), tenting area (154. 0 ± 42.0 mm2 vs. 143.9 ± 45.4 mm2, p NS), tenting height (6.9 ± 1.9 mm vs. 7.0 ± 1.8, p NS), tenting angle (131° ± 11 vs. 130° ± 10°)]. Likewise, LVESD, LVEF, LA volume, and RA volumes pre to post PVI favorably improved in the R group but not in NR (table 1).

**Table 1:** 

Cardiac MRI parameters	Responders to PVI (n=76)		P Value	Non-responders to PVI (n=18)		P Value
	PRE PVI	POST PVI		PRE PVI	POST PVI	

LAA VOLUME (ml)	3.3±1.7	3.1±1.8	NS	3.0±2.0	4.0±1.8	<0.05
LAA Os (mm)	19±4	18±4	NS	18±4	20±4	<0.05
LA volume Index (ml/m/2)	103±32	89±29	<0.01	95±29	83±37	NS
LVEF %	57±10	60±6	<0.001	59±9	60±8	NS
RA Volume Index (ml/m/2)	67±22	59±20	<0.001	45±19	54±18	NS
MR Severity (0 to 4+)	0.78	.51	<0.05	1.06	0.67	NS
LVEDD (mm)	52±5	52±5	NS	50±6	52±6	NS
LVESD (mm)	35±6	33±6	<0.01	35±6	35±6	NS

## Conclusions

Long-term success rate in AF patients following PVI remains suboptimal. In those with successful and durable maintenance of NSR, ventricular and atrial (LA and LAA) reverse remodeling demonstrated by 3D CMR occurs that is matched with marked improvements in mitral regurgitation and mitral apparatus likely begetting continued maintenance of NSR.

## Funding

Internal.

